# Genetic Variation in *ZmPAT7* Contributes to Tassel Branch Number in Maize

**DOI:** 10.3390/ijms23052586

**Published:** 2022-02-26

**Authors:** Honghui Guan, Xiaojing Chen, Kailiang Wang, Xuyang Liu, Dengfeng Zhang, Yongxiang Li, Yanchun Song, Yunsu Shi, Tianyu Wang, Chunhui Li, Yu Li

**Affiliations:** Institute of Crop Sciences, Chinese Academy of Agricultural Sciences, Beijing 100081, China; guanhonghui9202@163.com (H.G.); cxiaojing0918@163.com (X.C.); 18394821445@163.com (K.W.); liuxuyang@caas.cn (X.L.); zhangdengfeng@caas.cn (D.Z.); liyongxiang@caas.cn (Y.L.); songyanchun@caas.cn (Y.S.); shiyunsu@caas.cn (Y.S.); wangtianyu@caas.cn (T.W.)

**Keywords:** maize (*Zea mays* L.), tassel branch number (TBN), *ZmPAT7*, protein S-acyltransferases (PAT), expression quantitative trait locus (eQTL)

## Abstract

Tassel branch number (TBN) is one of the important agronomic traits that contribute to the efficiency of seed production and has been selected strongly during the modern maize breeding process. However, the genetic mechanisms of TBN in maize are not entirely clear. In this study, we used a B73 × CML247 recombination inbred lines (RILs) population to detect quantitative trait loci (QTLs) for TBN. A total of four QTLs (*qTBN2a*, *qTBN2b*, *qTBN4*, and *qTBN6*) and six candidate genes were identified through expression analysis. Further, one of the candidates (GRMZM2G010011, *ZmPAT7*) encoding an S-acyltransferase was selected to validate its function by CRISPR-Cas9 technology, and its loss-of-function lines showed a significant increase in TBN. A key SNP(−101) variation in the promoter of *ZmPAT7* was significantly associated with TBN. A total of 17 distant eQTLs associated with the expression of *ZmPAT7* were identified in expression quantitative trait loci (eQTL) analysis, and *ZmNAC3* may be a major factor involved in regulating *ZmPAT7*. These findings of the present study promote our understanding of the genetic basis of tassel architecture and provide new gene resources for maize breeding improvement.

## 1. Introduction

Maize is a monoecious cross-pollination crop. The male inflorescence (tassel) provides pollen for hybridization, which is important for maize grain production. Tassel size and weight have been reduced during modern breeding improvement [1,2]. Smaller tassels reduce the plant’s energy requirements for general maintenance and make more energy available for grain production [3]. On the other hand, tassel with sufficient pollens directly contributes to the efficiency of seed production and hybrid yield performance [4]. Therefore, an optimal tassel architecture is needed that can produce sufficient pollen and transform more energy into the kernel. As a major component of tassel architecture, tassel branch number (TBN) is significantly negatively correlated with grain yield, and the reduction in TBN has been experienced strong selection during the modern maize breeding process [5].

TBN is a complex quantitative trait controlled by multigenes. A large number of quantitative trait loci (QTLs)/quantitative trait nucleotides (QTNs) for TBN have been mapped in different mapping populations. Zhao et al. (2017) detected 11 QTLs using two F_2:3_ populations and identified 13 meta-QTLs (mQTLs) by integrating QTLs reported previously [6]. Li et al. (2018) summarized 100 QTLs and 360 QTNs for TBN, identified in several previous studies, and found that these QTLs and QTNs were mainly located at 20 genomic regions [7]. Recently, Wang et al. (2018) detected six minor effect QTLs for TBN using a Zheng58 × Chang7-2 recombination inbred lines (RILs) population with super-high-density genotyping [8]. Liu et al. (2019) detected seven QTLs using an F_2:3_ population that generated from the cross between Lv28 and H082183 [9]. Wang et al. (2019) detected 51 QTNs and 19 QTLs associated with TBN in an association mapping population and a doubled haploid (DH) population, respectively [10]. Notably, a nested association mapping (NAM) population was used to identify 39 QTLs and 325 QTNs for TBN by joint linkage analysis and genome-wide association studies (GWAS) [11]. Furthermore, Wu et al. (2016) integrated two NAM populations (a NAM from the US and a NAM from China) and identified 63 QTLs and 549 QTNs for TBN [12]. Identification of those QTLs is beneficial to elucidating the genetic basis and facilitating gene cloning for TBN in maize.

Some tassel architecture-related genes were cloned based on maize mutants previously. For example, *RAMOSA2* (*RA2*), and *RAMOSA3* (*RA3*), which function in the *ramosa* pathway, regulate the identity and determinacy of inflorescence axillary meristems. Additionally, those *ramosa* mutants show an increase in tassel branch number [13,14]. The two fasciated ear mutants, *fasciated ear2* (*fea2*) and *fasciated ear3* (*fea3*)*,* also showed a larger tassel branch number. *FEA2* and *FEA3* encode membrane-localized leucine-rich repeat receptors and involve in the *CLAVATA* pathway [15,16]. *UNBRANCHED2* (*UB2*), *UB3*, and *TSH4* all encode SBP-box genes and function as redundant factors that limit the rate of cell differentiation to the lateral primordia. Loss-of-function mutations in *ub2*, *ub3*, and *tsh4* result in smaller TBN [17,18]. *Barren inflorescence2* (*bif2*) mutation has a reduced number of branches and spikelets in tassels. *BIF2* encodes a serine–threonine protein kinase, which is modified the transition from inflorescence meristem (IM) to spikelet pair meristem (SPM) and indirectly affected the development of branch meristems [19,20]. However, because of negative pleiotropy, these genes cloned by mutants are difficult to use in maize breeding. Recently, a natural variation related to tassel branch number was identified in the 5′-UTR (untranslated region) of *Q^Dtbn1^*. *Q^Dtbn1^* encodes a Kelch repeat-containing F-box protein, negatively regulates TBN with a dominant model, and participates in the ABA signaling pathway [21]. Despite these studies, there are still very few genes cloned through natural variation.

In this study, a B73 × CML247 RILs population was used to detect QTLs for TBN. Four QTLs were detected and colocated with the TBN-related associated loci that were reported by Wu et al. (2016), using two NAM populations to identify candidate genes [12]. *ZmPAT7* was identified as a major candidate gene through bioinformatics and expression analysis. *ZmPAT7* negatively regulated TBN, and its knockout lines had more TBN. A key SNP variation in the promoter of *ZmPAT7* may confer the expression of *ZmPAT7* and shows significant association with TBN. Moreover, 17 distant eQTLs associated with the expression of *ZmPAT7* were identified in expression quantitative trait loci (eQTL) analysis.

## 2. Results

### 2.1. QTL Mapping and Identification of Candidate Genes for TBN

**Figure 1:**
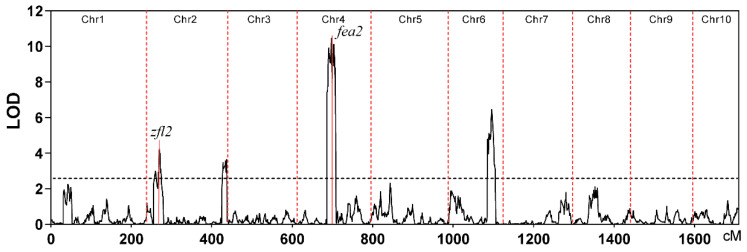
QTLs detected for TBN in the B73 × CML247 RILs population. *zfl2* and *fea2* were colocated with *qTBN2a* and *qTBN4*, respectively.

The two inbred lines, B73 and CML247, display great differences in tassel branch number. CML247 plants had more TBN than B73 plants, and the B73 × CML247 RIL population also revealed considerable variations in TBN (Figure S1a). Therefore, we used the RIL population to conduct subsequent QTL mapping. A total of four QTLs were detected above a cutoff of LOD > 2.5, including two QTL (*qTBN2a* and *qTBN2b*), one QTL (*qTBN4*), and one QTL (*qTBN6*) on chromosomes 2, 4, and 6, respectively (Figure 1, Table 1). Individual QTLs explained phenotypic variation ranging from 5.21% to 16.22%, and *qTBN4* showed the largest effect among the four QTLs (Table 1). The number of genes in the interval of QTLs varied from 217 to 2510 (Table 1). We further checked whether the known cloned genes regulating the development of male inflorescence were located in QTL regions. We found that *FASCIATED EAR2* (*FEA2*), involved in the *CLAVATA* pathway to control the size of the inflorescence meristem, was colocated with *qTBN4*. *zfl2*, whose mutant exhibited disruptions of floral organ identity and patterning, and defects of inflorescence architecture, and in the vegetative to reproductive phase transition [22], they were colocated with *qTBN2a*.

Previously, Wu et al. (2016) performed a genome-wide association study using the CN-NAM and US-NAM populations for tassel architecture and identified a total of 549 QTNs for TBN [12]. It was found that 25 among the 549 QTNs were colocated in the four QTLs detected in this study, including 15 QTNs colocated with *qTBN2a*, 4 QTNs colocated with *qTBN2b*, 5 QTNs colocated with *qTBN4*, and 1 QTN colocated with *qTBN6*. Subsequently, we identified a total of 36 candidate genes underlying TBN (Table 2). Furthermore, we compared the expression of those candidate genes in shoot apical meristem (SAM) between the two parents, i.e., B73 and CML247 (Figure S1b). Six candidate genes (GRMZM2G302701, GRMZM5G897776, GRMZM2G162266, GRMZM2G163067, GRMZM2G010011, and GRMZM2G423636) showed high expression and also differential expression between B73 and CML247, which might play a role in regulating maize TBN. The three candidate genes, i.e., GRMZM2G162266, GRMZM2G163067, and GRMZM2G423636, encode uncharacterized proteins. GRMZM2G010011 encodes an S-acyltransferase. GRMZM2G302701 encodes a nucleolar preribosomal-associated protein 1. GRMZM5G897776 encodes a starch synthase 3 involved in starch biosynthesis.

### 2.2. ZmPAT7 Negatively Controls Tassel Branch Number in Maize

**Figure 2:**
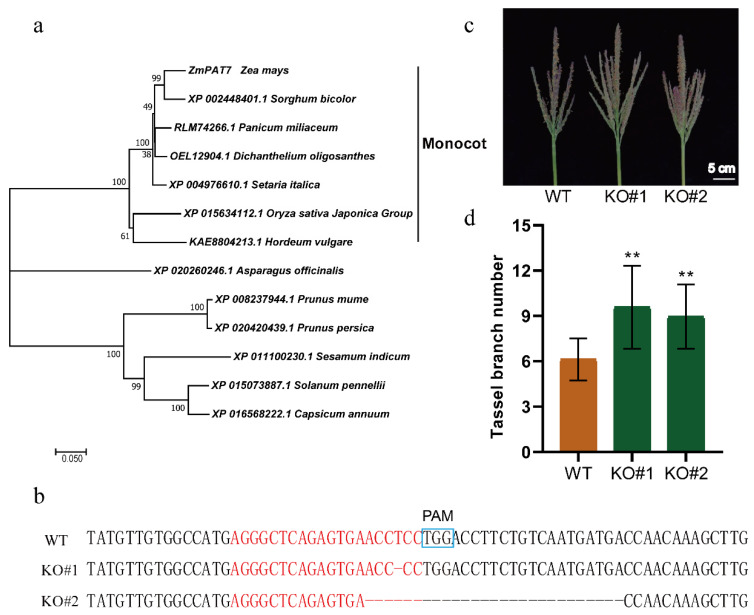
Phylogenetic tree of PAT proteins and the TBN performance in *ZmPAT7* CRISPR/Cas9 mutants. (**a**) phylogenetic analysis of ZmPAT7 and 12 PAT proteins from other plants; (**b**) knockout of *ZmPAT7* by CRISPR/Cas9 technology, the blue box is protospacer adjacent motif (PAM) sequence, the red font is single guide sequence (20 bp sequence adjacent to PAM) targeting *ZmPAT7* in inbred line KN5585. Image (**c**) and statistics (**d**) show the TBN differences between wild-type (WT) and CRISPR-knockout (KO#1 and KO#2) plants. Data are represented as mean ± SD; ** indicates significant difference at 0.01 level by two-sided *t*-test.

Previous studies suggested that S-acyltransferases play an important role in regulating plant architecture in plants [23,24,25,26]. Therefore, we focused on GRMZM2G010011 to further validate its function. Previously, 38 Protein S-acyltransferases (PAT) genes have been identified in the maize genome, and GRMZM2G010011 was named *ZmPAT7* [27]. ZmPAT7 harbors a conserved Asp–His–His–Cys (DHHC) domain that is representative of S-acyltransferases and has four transmembrane motifs (Figure S2a,b). Phylogenetic analyses of ZmPAT7 with high homology protein from other plants showed that ZmPAT7 was closely related with PAT proteins in monocot (Figure 2a). To confirm the function of *ZmPAT7* underlying TBN, we obtained two knockout lines (KO#1 and KO#2) using CRISPR/Cas9 technology. Gene editing of *ZmPAT7* was screened by PCR amplification and Sanger sequencing of the target region. KO#1 carrying a 1 bp deletion and KO#2 carrying a 27 bp deletion were used for subsequent analysis (Figure 2b). Compared with the wild-type line, the two loss-of-function lines of *ZmPAT7* showed a significant increase in TBN (Figure 2c,d).

### 2.3. Natural Alleles of ZmPAT7

**Figure 3:**
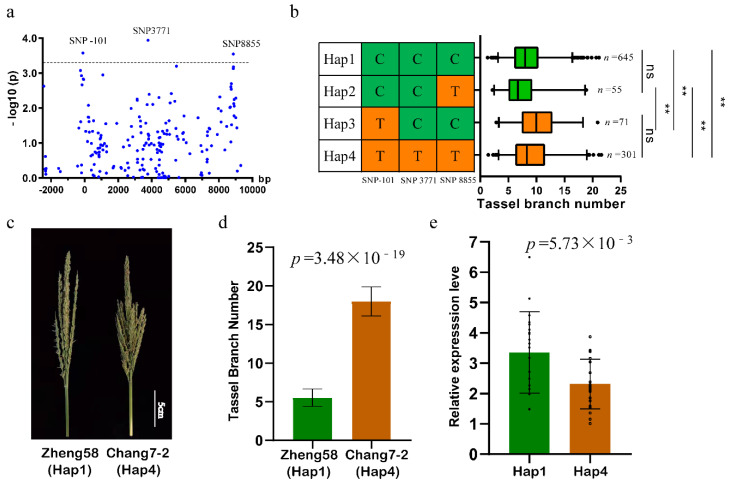
Genetic variations in the *ZmPAT7* promoter are linked to TBN: (**a**) association analysis of the genetic variation in *ZmPAT7* with TBN; (**b**) the TBN values of inbred lines of the four haplotypes are displayed in the box plot. Here, n denotes the number of inbred lines belonging to each haplotype group. Statistical significance was determined using a two-sided *t*-test. ** indicates significant difference at 0.01 level; ns indicates no significant difference. Image (**c**) and statistics (**d**) show the TBN differences between Zheng58 and Chang7-2. Values are represented as mean ± SD, and the *p* values of two-sided *t*-test are shown; (**e**) expression analysis of *ZmPAT7* between the 20 lines with Hap1 and the 20 lines with Hap4.

To identify key variations in *ZmPAT7* associated with TBN, we obtained the polymorphic variants in a ~12.5 kb genomic region, covering the promoter and gene body of *ZmPAT7*, from 1604 resequenced maize inbred lines. We identified 200 polymorphic variants (SNPs and indels) (Figure 3a) and found that three SNPs (−101, 3771, and 8855 bp) were significantly associated with TBN. The three SNPs were located in the promoter, the fifth intron region, and downstream of the gene body, respectively. Based on the three significant variants, we classified the 1604 maize genotypes into 4 haplotype groups (Figure 3b). The lines with Hap1 or Hap2 have lower TBN than those with Hap3 or Hap4, and no significant differences were observed between lines with Hap1 and Hap2 or between lines with Hap3 and Hap4. Therefore, we designated Hap1 or Hap2 as a favorable haplotype, which is consistent with the haplotype groups classified only by SNP (−101). The SNP (−101) may be a key variant in regulating TBN. For example, the inbred lines Zheng58 and Chang7-2, two parents of hybrid Zhengdan958 widely planted in China, had significant differences in TBN (Figure 3c,d). Zheng58 with Hap1 had a smaller TBN (5.5 ± 1.1) than Chang7-2 with Hap4 (18 ± 1.9). Furthermore, we chose 20 lines with Hap1 and 20 lines with Hap4 to conduct gene expression analysis. *ZmPAT7* exhibited higher expression in low TBN lines with Hap1 than that in high TBN lines with Hap4 (Figure 3e). These results suggested that the expression of *ZmPAT7* may contribute to the phenotypic variation in maize tassel branch number.

### 2.4. Expression Quantitative Trait Loci Analysis of ZmPAT7

**Figure 4:**
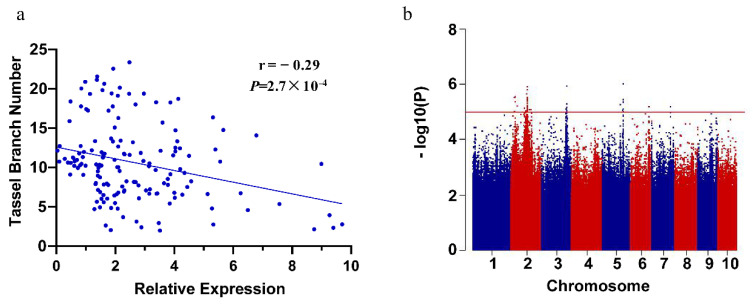
eQTL analysis for *ZmPAT7*: (**a**) the expression of *ZmPAT7* at the V5 stage negatively correlated with TBN (**b**) Manhattan plot for eQTL analysis, with the suggestive threshold of 1.0 × 10^−5^.

We used 223 inbred lines with large phenotypic variations in TBN (from 1.97 to 23.37) (Table S1, Figure S3) to analyze the expression level of *ZmPAT7*. The expression of *ZmPAT7* showed a considerable variation and negatively correlated with TBN (r = −0.29, *p* = 2.7 × 10^−4^) (Figure 4a). Association analysis was conducted using the expression of *ZmPAT7* as phenotypic value and a total of 49 significantly associated SNPs were identified (Figure 4b). Based on the significant SNPs, a total of 17 eQTLs associated with the expression of *ZmPAT7* were identified. These included nine on chromosome 2, three on chromosome 3, three on chromosome 5, one on chromosome 6, and one on chromosome 7 (Table 3). All the 17 eQTLs were defined as “distant eQTLs” following the previous method proposed by Fu et al. (2013) and Pang et al. (2019) [28,29]. In total, 44 genes were found in LD distance (200 kb) adjacent to the SNPs (Table 3), among which some transcription factor genes, e.g., Zm00001d038207 (*ZmNAC3*), may be involved in regulating *ZmPAT7*. Interestingly, we also found an SNP (−274, T/A) in the promoter of *ZmPAT7* between B73 and CML247, which results in the *cis*-element NAC motif change. The NAC motif (TCTTGACC) existed in B73, while the NAC core promoter element was absent in CML247 (Figure S4). These results suggested that *ZmNAC3* may be a major factor that mediated the expression of *ZmPAT7*.

## 3. Discussion

In this study, we mapped four QTLs for TBN by using a high-quality recombination map of the B73 × CML247 RIL population. In previous studies, all identified QTLs were colocated with the QTLs/QTNs detected in other populations [6,9,11,12]. *qTBN2a* mapped at 7.85–14.72 Mb interval on chromosome 2 in our study was also reported previously. For example, *Q32_CN-NAM_* and *Q46_US-NAM_* identified by Wu et al. (2016) using joint linkage analysis for TBN [12] were colocated with *qTBN2a*. Zhao et al. (2017) also identified an mQTL2-1 on chromosome 2 using meta-QTL analysis [6], which overlapped with *qTBN2a* in our study. In addition, a tassel dry weight (TW) related QTL, *qTW2_1*, was also colocated with *qTBN2a* [9]. *Q50_US-NAM_* and four QTNs reported by Wu et al. (2016) [12] were colocated with *qTBN2b*, and *Q44_CN-NAM_* reported by the same study was colocated with *qTBN6*. Eight QTNs and five QTNs reported by Brown et al. (2011) [11] and Wu et al. (2016) [12], respectively, fell in the interval of *qTBN4*. Furthermore, some maize inflorescence developmental genes were located in those QTLs detected in this study. For example, *zfl2*, regulating the development of inflorescence architecture, was colocated with *qTBN2a*. In addition, *fea2*, controlling the size of the inflorescence meristem, was colocated with *qTBN4*. These comparisons suggested that the QTLs detected in the present study were important genomic regions controlling TBN.

A large number of QTLs for TBN have been identified by using bi-parental populations [6,8,9,30,31,32,33]. These QTLs have generally large genomic intervals, which is difficult to find candidate genes quickly. The interval of QTLs in our study is also large, containing many genes (Table 1). On another front, many QTNs for TBN were identified using GWAS [11,12,34]. Combining GWAS and linkage mapping would be beneficial to quickly identify candidate genes. For example, Wang et al. (2019) detected 55 candidate genes according to the colocated loci between QTNs and QTLs [10]. Wu et al. (2016) detected 113 male inflorescence-related candidate genes by integrating the results of joint-linkage mapping and GWAS [12]. To identify candidate genes, we integrated our QTL results and the QTNs of GWAS from the US-NAM and CN-NAM populations reported by Wu et al. (2016) [12]. A total of 25 QTNs were colocated with the 4 QTLs obtained in this study, and 36 candidate genes were then identified.

Transcriptome data can be used in narrowing the range of candidate genes for specific traits. Thus, we used the expression data in B73 and CML247 shoot apical meristem (SAM) from https://qteller.maizegdb.org/ (accessed on 18 May 2020) [35] for identifying the most possible candidate genes. Six candidate genes (GRMZM2G302701, GRMZM5G897776, GRMZM2G162266, GRMZM2G163067, GRMZM2G010011, and GRMZM2G423636) were identified to further explore the regulation mechanisms of TBN, among which GRMZM2G010011 (*ZmPAT7*) encodes an S-acyltransferase.

S-acylation is a reversible post-translational modification process occurring in eukaryotes that regulates tracking, regulation, signaling, membrane association, and target protein functions [36]. Several studies have reported that S-acyltransferases encoded by the PAT gene family play an important role in regulating plant architecture and other traits in plants. For instance, *OsPAT15* regulates plant architecture by altering the tiller in rice [24], and overexpression of *OsPAT15* results in increased branch and seed yield in *Brassica napus* [37]. Knockout lines of *PbPAT14* exhibited the dwarf yellowing phenotype, and the function of *PbPAT14* was related to the ABA pathway [25]. In addition, Hemsley et al. (2005) reported an S-acyltransferase gene in *Arabidopsis*, *Tip Growth Defective 1* (*TIP1*)*/PAT24*, which exhibits impairment in root hair elongation and defective growth of pollen tubes [23]. Zhang et al. (2020) reported that *ZmTIP1*, which encodes a functional S-acyltransferase, plays a positive role in regulating the length of root hairs and drought tolerance in maize [26]. Two homologous proteins, PAT13 and PAT14, are cooperatively involved in regulating precocious leaf senescence in *Arabidopsis* [38,39]. PAT10 is localized on Golgi and tonoplast membranes, and those mutations performed pleiotropic defects in plant development [40,41]. PAT4 was also reported to mediate root hair growth in *Arabidopsis* [42]. *AtPAT21*, another PAT reported recently, participates in both male and female gametogenesis [43]. Although ZmPAT7 harbors a conserved DHHC domain that is representative of S-acyltransferases (Supplementary Figure S2a,b), and phylogenetic analyses of ZmPAT7 showed that it was highly conserved in monocots, functions of these S-acyltransferase proteins that are close to ZmPAT7 are still unknown.

In our study, the two loss-of-function lines of *ZmPAT7* showed significant increases in TBN (Figure 2c,d). *ZmPAT7* exhibited higher expression in low TBN lines than that in high TBN lines (Figure 3d), suggesting that the expression of *ZmPAT7* negatively regulated TBN in maize. We identified a key variant SNP (−101) in the *ZmPAT7* promoter which caused the change in expression level and was further verified in the inbred lines of Zheng58 and Chang7-2 (Figure 3b,c). Additionally, the sequences of *ZmPAT7* promoter in B73 and CML247 also showed several variants (Supplementary Figure S4), e.g., the SNP (−157, A/C) resulting in the ERF element difference, the SNP(−274, T/A) in the *cis*-element NAC motif change, and the SNP(−433, G/T) in the MYB element difference. Taken together, we speculated that *ZmPAT7* plays an important role in natural variations in TBN in maize.

## 4. Materials and Methods

### 4.1. Plant Materials

The B73 × CML247 RILs population containing 193 lines was used for QTL mapping. A large association population containing 1604 maize inbred lines comprising 1227 from China, 344 from the United States, and 33 from other countries [44] was used for candidate gene association analysis. Furthermore, we chose 223 inbred lines that showed considerable variations in TBN from the association panel to conduct expression assay and eQTL analysis. To detect the expression of *ZmPAT7*, we grew the 223 inbred lines in a field in Beijing, China, in the summer of 2019, and sampled the leaf at the V5 stage.

### 4.2. QTL Mapping and Identification of Candidate Genes

The phenotypic data of TBN for the US-NAM RILs were collected in eight environments as described by Brown et al. (2011) [11], which were transformed using the Box–Cox function in R software, and used to calculate best-unbiased predictor (BLUP). The BLUP values of TBN in B73 × CML247 RILs were used for QTL mapping. A high-quality recombination map containing 2183 bin markers of the B73 × CML247 RILs was reported by Li et al. (2015) [45]. The total linkage distance of the genetic map is 1708.44 cM, and the average interval between adjacent bins was 0.78 cM [45]. A total of 193 RILs were matched between the phenotype and genotype. QTL analysis was conducted using the WinQTLCart cartographer v2.5 software (North Carolina State University, Raleigh, NC, USA) with composite interval mapping (CIM) method [46], with a 1000 permutation test at 95% confidence level to determine the optimal log of odds (LOD) threshold values. Then, the 2-LOD drop method was applied for defining the QTL confidence interval. Then, we identified the colocated loci with the QTNs reported by Wu et al. (2016) [12]. Genes falling within 50 kb of colocated loci were considered candidate genes. Furthermore, the expression data in B73 and CML247 shoot apical meristem (SAM) of candidate genes was extracted from https://qteller.maizegdb.org/ (accessed on 18 May 2020) [35] and used to find differential expression genes between B73 and CML247. The genes with high expression in SAM and the ratio of expression between B73 and CML247 greater than 1.5 (B73/CML247 > 1.5 or CML247/B73 > 1.5) were considered candidate genes.

### 4.3. Identification of the CRISPR/Cas9 Mutants of ZmPAT7

The CRISPR/Cas9 mutants of *ZmPAT7* were ordered from http://www.wimibio.com/maize.html (accessed on 5 May 2019) and the methods of vector construction, genetic transformation for creating CRISPR/Cas9 mutants were reported by Liu et al. (2020) [47]. Briefly, the single guide RNA (sgRNA) sequence is AGGGCTCAGAGTGAACCTCC. The vector pCPB-*ZmUbi-hspCas9* was first linearized by *Hin*dIII. Separately, *ZmU6* and the sgRNA scaffold of *ZmPAT7* were amplified through overlapping PCR with a homologous arm. Additionally, homologous arms that match linearized pCPB-*ZmUbi-hspCas9* were also added to the insertion fragment in the overlap PCR. Finally, the gene-specific insertion fragments were incorporated into the pCPB-*ZmUbi-hspCas9* vector. Additionally, the maize inbred line KN5585 was then used for *Agrobacterium tumefaciens*-mediated transformation of immature embryos. We planted the T1 lines in the field to identify the homozygous positive lines. Genomic editing of *ZmPAT7* was screened by PCR amplification and Sanger sequencing of the target region. The PCR primers were cr-F: CAATCTTGACAAAGAAACC and cr-R: TTGAAATGATAGAACGCAC. The T2 homozygous positive lines and its wild-type line KN5585 were phenotyped at Sanya (18° N, 109° E) in the winter of 2020. The experiment in the field followed a randomized block design, with three replicates.

### 4.4. Candidate Gene-Based Association Analysis

The phenotypic data of TBN for the 1604 inbred lines were identified at five locations in 2018 and 2019, which were used to calculate the best-unbiased predictor (BLUP). The polymorphic variants in a ~12.5 kb region covering 2.5 kb upstream, 9.5 kb gene body of *ZmPAT7* and 0.5 kb downstream region were extracted from 1604 inbred lines resequenced data [44]. Those variants with minor allele frequency (MAF) over 5% were identified and used for candidate gene-based association analysis. Association analysis was performed using a mixed linear model (MLM) in Tassel v3.0 (Cornell University, Ithaca, NY, USA), with the population structure and kinship analyzed in Tassel v5.0 [48]. A Bonferroni-corrected significant association threshold (*p* ≤ 0.1/200 = 5.0 × 10^−4^) was used to determine the significantly associated variants.

### 4.5. Expression Quantitative Trait Loci Analysis

The leaves at V5 of 223 inbred lines were isolated for qRT–PCR. A total of 8,974,340 SNPs was identified with a minor allele frequency (MAF) > 0.05. The Kinship (K) matrix of pairwise genetic distances was calculated by EMMAX and was used as the variance-covariance matrix of the random effects for association analysis. The association analysis using a mixed linear model (MLM) was performed with the EMMAX software package. The suggestive threshold was 1.0 × 10^−5^, which was used to identify significantly associated signals. The significant SNPs were defined as candidate eQTLs, and if there were more than two SNPs in a 5 kb region, the SNPs were regarded as a cluster, and the most significant SNPs in the clusters were considered as candidate eQTLs. If two candidate eQTLs of a single gene were under LD (r^2^ > 0.1), the less significant eQTL was removed. The eQTLs were divided into two types using 20 kb as a cutoff to define “local eQTLs” versus “distant eQTLs”. The methods mentioned above were similar to those in previous studies [28,29].

### 4.6. RNA Isolation and Quantitative Reverse Transcription PCR (qRT–PCR)

Total RNAs were extracted from plant tissues using the Plant Total RNA Isolation Kit (Genebetter, Beijing, China). First-strand cDNA was synthesized using the Uni One-Step gDNA Removal and cDNA Synthesis SuperMix Kit (TranScript, Beijing, China). Quantitative real-time PCR (qRT–PCR) was performed using the Taq Pro Universal SYBR qPCR Master Mix (Vazyme, Nanjing, China) with a QuanStudio 3 Real-Time PCR System cycler (Applied Biosystems, San Francisco, CA, USA). The maize *GAPDH* gene was used as the internal control, and the relative expression was calculated using the 2^−ΔΔCt^ method. The PCR conditions consisted of an initial denaturation step at 95 °C for 30 s, followed by 40 cycles at 95 °C for 10 s, and 60 °C for 30 s. The primers of *ZmPAT7* and *GAPDH* were qPAT7 (qPAT7-f: CCAGTATGAGGCAGACAGTGA, qPAT7-R: TGGATGGTGCTCGGCTATG) and qGAPDH (qGAPDH-F: CCCTTCATCACCACGGACTAC qGAPDH-R: AACCTTCTTGGCACCACCCT), respectively.

### 4.7. Sequence Blast and Phylogenetic Analysis

The sequences of *ZmPAT7* promoter in B73 and CML247 were obtained from maizeGDB (https://www.maizegdb.org/, accessed on 18 May 2021). Sequence alignment was conducted using Tbtools (a Toolkit for Biologists integrating various biological data-handling tools) [49]. Homologs of ZmPAT7 were identified using the BLASTP (https://blast.ncbi.nlm.nih.gov/, accessed on 10 October 2021). The phylogenetic tree was conducted using the neighbor-joining method in MEGA7.0 (https://www.megasoftware.net/, accessed on 10 October 2021) with 1000 replicate bootstrap, and the following parameters: p-distance mode, pairwise deletion. *Cis*-acting elements on the promoter were predicted using PlantPAN 3.0 [50].

## Figures and Tables

**Table 1:** List of QTL for TBN in the B73 × CML247 RILs population.

QTL	Chr	Peaks (cM)	Interval (cM)	LOD	Interval (Mb)	Add	R^2^ (%)	Number of Genes in the Interval
*qTBN2a*	2	30.12	17.25–37.37	4.24	7.85–14.72	−0.56	6.08	571
*qTBN2b*	2	192.7	187.05–199.07	3.69	233.63–236.44	0.56	5.21	217
*qTBN4*	4	68.09	61.14–77.39	10.47	58.51–134.62	0.93	16.22	2510
*qTBN6*	6	104.6	99.09–109.45	6.47	152.15–156.88	−0.7	9.53	413

**Table 2:** List of colocated SNPs and the annotation of candidate genes.

QTL	Tag SNPs	Chr	Pos (bp)	*p* Value	Candidate Genes That near the Tag SNPs
*qTBN2a*	S2_8809018	2	8,809,018	3.77 × 10^−1^^0^	GRMZM2G002559; GRMZM2G302701; GRMZM2G302712
	S2_8889278	2	8,889,278	3.31 × 10^−^^9^	GRMZM5G897776
	S2_9443046	2	9,443,046	1.09 × 10^−1^^0^	GRMZM2G080041; GRMZM2G080054
	S2_11829845	2	11,829,845	5.70 × 10^−1^^0^	GRMZM2G392125; GRMZM2G091118; GRMZM2G090872
	S2_12687587	2	12,687,587	5.61 × 10^−1^^1^	GRMZM2G149556
	S2_12691259	2	12,691,259	7.73 × 10^−1^^0^	GRMZM2G149556
	S2_12798388	2	12,798,388	9.40 × 10^−1^^1^	GRMZM2G024898
	S2_12898426	2	12,898,426	2.16 × 10^−1^^1^	GRMZM2G463280; GRMZM2G463267; GRMZM2G162266
	S2_13299879	2	13,299,879	4.32 × 10^−1^^0^	GRMZM2G038722; GRMZM2G038714; GRMZM2G342039
	S2_13636982	2	13,636,982	4.49 × 10^−1^^0^	
	S2_13638186	2	13,638,186	8.50 × 10^−^^9^	GRMZM2G090332
	S2_14264763	2	14,264,763	3.38 × 10^−^^9^	GRMZM2G163067
	S2_14267757	2	14,267,757	3.8 × 10^−1^^0^	GRMZM2G163067
	S2_14467324	2	14,467,324	1.38 × 10^−1^^0^	GRMZM2G146866; GRMZM2G146847; GRMZM2G010011
	S2_14541295	2	14,541,295	2.43 × 10^−1^^0^	GRMZM2G314396; GRMZM2G017197; GRMZM2G314386; GRMZM2G314412
*qTBN2b*	S2_233632388	2	233,632,388	5.21 × 10^−1^^0^	GRMZM2G176347; GRMZM2G176375
	S2_234531325	2	234,531,325	1.94 × 10^−1^^0^	GRMZM2G423640; GRMZM2G423636
	S2_234707306	2	234,707,306	1.46 × 10^−^^9^	GRMZM2G324507
	S2_235802795	2	235,802,795	1.03 × 10^−1^^0^	GRMZM2G347995
*qTBN4*	S4_66857201	4	66,857,201	3.35 × 10^−1^^0^	GRMZM2G399421
	S4_66908736	4	66,908,736	1.39 × 10^−1^^0^	GRMZM2G030628
	S4_71986141	4	71,986,141	7.14 × 10^−1^^2^	GRMZM2G448456
	PZE-104061279	4	120,847,621	3.38 × 10^−^^6^	
	S4_122747978	4	122,747,978	1.53 × 10^−1^^2^	
*qTBN6*	S6_153206873	6	153,206,873	2.97 × 10^−1^^2^	GRMZM2G359892

**Table 3:** List of eQTL for *ZmPAT7* and the annotation of candidate genes.

Code of eQTL	Peak_SNP	*p*_Value	V4_Gene_Id	Annotation
eQTL-1	2:26,517,652	3.05 × 10^−6^	Zm00001d002917	RNA-binding region RNP-1
eQTL-2	2:33,010,637	4.51 × 10^−6^	Zm00001d003123	NA
			Zm00001d003124	DNA glycosylase superfamily protein
			Zm00001d003125	Uncharacterized protein
eQTL-3	2:52,125,742	6.25 × 10^−6^		
eQTL-4	2:104,153,316	7.62 × 10^−6^	Zm00001d004336	NA
eQTL-5	2:115,870,588	9.34 × 10^−6^	Zm00001d004509	Terpene synthase 7
			Zm00001d004512	Transcription factor E2FC
			Zm00001d004513	Bifunctional 3-phosphoadenosine 5-phosphosulfate synthetase 2
eQTL-6	2:122,415,924	2.26 × 10^−6^	Zm00001d004619	NA
eQTL-7	2:131,987,662	1.27 × 10^−6^	Zm00001d004697	IAA27-auxin-responsive Aux/IAA family member
			Zm00001d004698	Serine–threonine-protein kinase-like protein CCR4
eQTL-8	2:147,699,071	8.28 × 10^−6^	Zm00001d004897	Basic-leucine zipper (bZIP) transcription factor family protein
eQTL-9	2:158,878,809	8.32 × 10^−6^	Zm00001d005112	SAC3 family protein B
eQTL-10	3:194,080,838	8.12 × 10^−6^	Zm00001d043273	SR protein related family member
			Zm00001d043274	Insulin-degrading enzyme-like 1 peroxisomal
			Zm00001d043275	Vacuole membrane protein KMS1
eQTL-11	3:200,375,447	9.92 × 10^−6^	Zm00001d043444	plant-specific domain TIGR01615 family protein expressed
			Zm00001d043445	Dihydroorotase
			Zm00001d043446	Uncharacterized protein
			Zm00001d043447	PP2A
			Zm00001d043449	RNA-binding S4 domain-containing protein
			Zm00001d043450	DNA-binding WRKY
			Zm00001d043451	Dynamin-related protein 5A
			Zm00001d043452	Hox2a protein
			Zm00001d043453	Early nodulin-like protein 3
			Zm00001d043454	Uncharacterized protein
eQTL-12	3:200,789,018	8.80 × 10^−6^	Zm00001d043461	ZCN12
			Zm00001d043462	Uncharacterized protein
			Zm00001d043463	Protein NRT1/PTR FAMILY 4.2
			Zm00001d043464	Oil body-associated protein 2A
			Zm00001d043465	50S ribosomal protein L13 chloroplastic
eQTL-13	5:143,694,885	5.49 × 10^−6^	Zm00001d016083	NA
			Zm00001d016084	Pre-mRNA-splicing factor 3
			Zm00001d016085	Putative uncharacterized protein
eQTL-14	5:163,991,516	8.74 × 10^−6^	Zm00001d016477	Eukaryotic aspartyl protease family protein
eQTL-15	5:170,985,132	1.02 × 10^−6^	Zm00001d016636	Putative uncharacterized protein
			Zm00001d016637	NA
eQTL-16	6:151,600,488	6.51 × 10^−6^	Zm00001d038205	MLO-like protein
			Zm00001d038206	Pentatricopeptide repeat-containing protein mitochondrial
			Zm00001d038207	*ZmNAC3*, NAC domain-containing protein 35
eQTL-17	7:145,022,165	6.56 × 10^−6^	Zm00001d021172	NA
			Zm00001d021173	Formin-like protein 1
			Zm00001d021176	Disease resistance protein RPM1
			Zm00001d021177	NA

## Data Availability

Not applicable.

## Supplementary Materials

The following supporting information can be downloaded at: https://www.mdpi.com/article/10.3390/ijms23052586/s1.
