# Supplementary material for: Genetic Variation in ZmPAT7 Contributes to Tassel Branch Number in Maize
Source: Int J Mol Sci. 2022 Feb 26;23(5):2586. doi: 10.3390/ijms23052586 (PMC8910302; doi:10.3390/ijms23052586)
Supplement: Supplementary file 1 [file ijms-23-02586-s001.zip › ijms-1613249-supplementary.pdf]

Table S1. Descriptive statistics of phenotype data.

Population	Range	Mean	SD	Skewness	Kurtosis	CV %	$H^{2\,b}$	Reference
B73 × CML247 RILs [a]	14.83–26.04	19.66	2.25	0.166	−0.335	11.44%	90.52%	[11]

a: BLUP values were used for Descriptive statistics.

b: The heritability is calculated using original data.

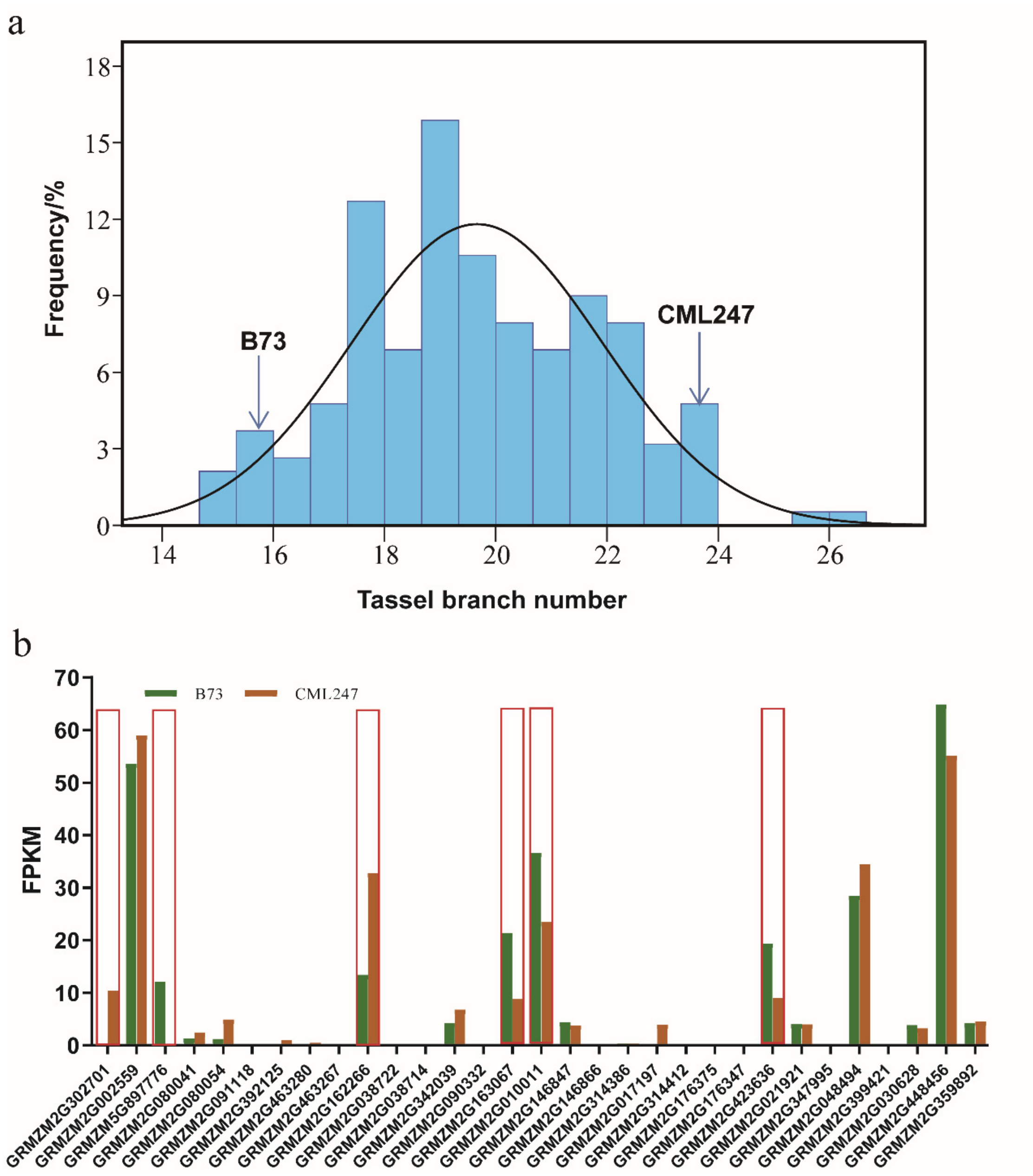

Figure S1. Phenotypic distribution of the B73×CML247 RIL population and expression analysis of candidate genes. **a**: Distributions of TBN in B73×CML247 RILs population. **b:** The expression of candidate genes in shoot apical meristem (SAM) between the two parents B73 and CML247. The red boxes refer to the differentially expressed genes between B73 and CML247.

a

b

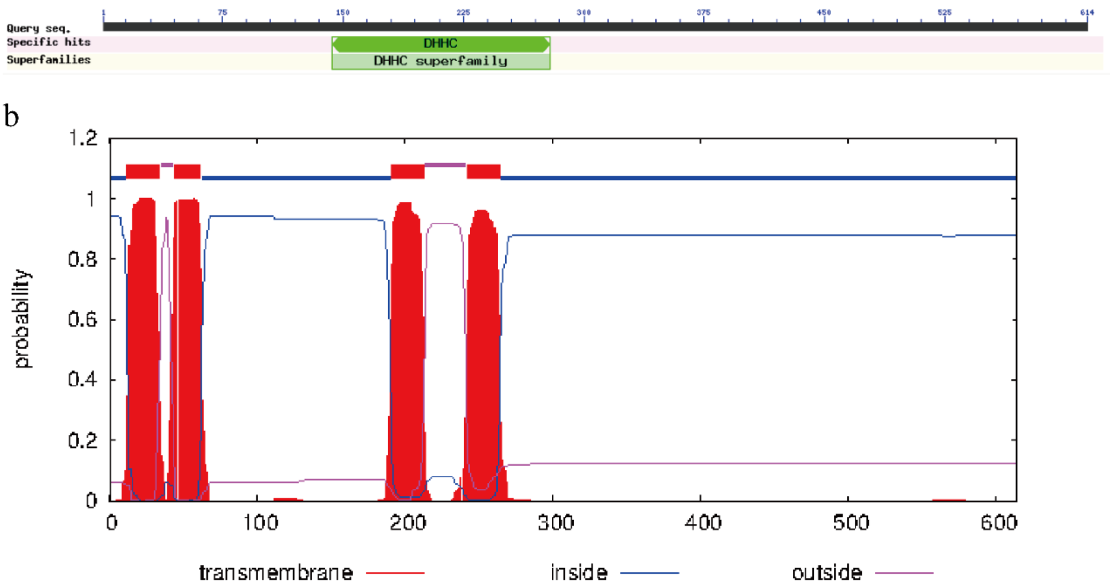

Figure S2. Prediction of conserved domain and transmembrane helices in *ZmPAT7*.
a: Prediction of conserved domain using NCBI Conserved Domain Search. **b**: Prediction of transmembrane helices using TMHMM-2.0.

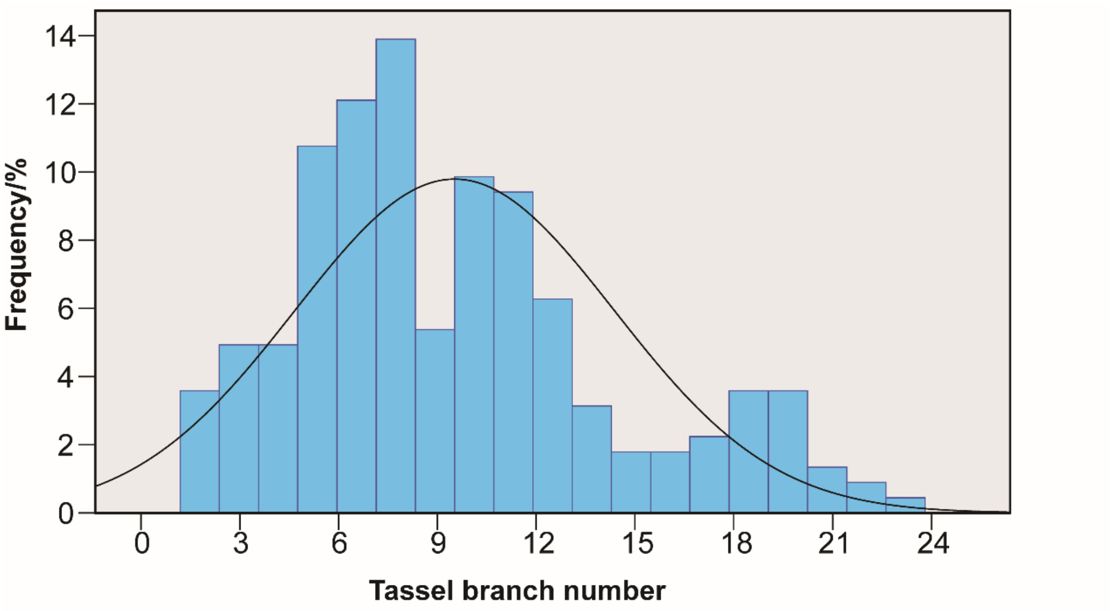

Figure S3. Phenotypic distribution of 223 inbred lines.

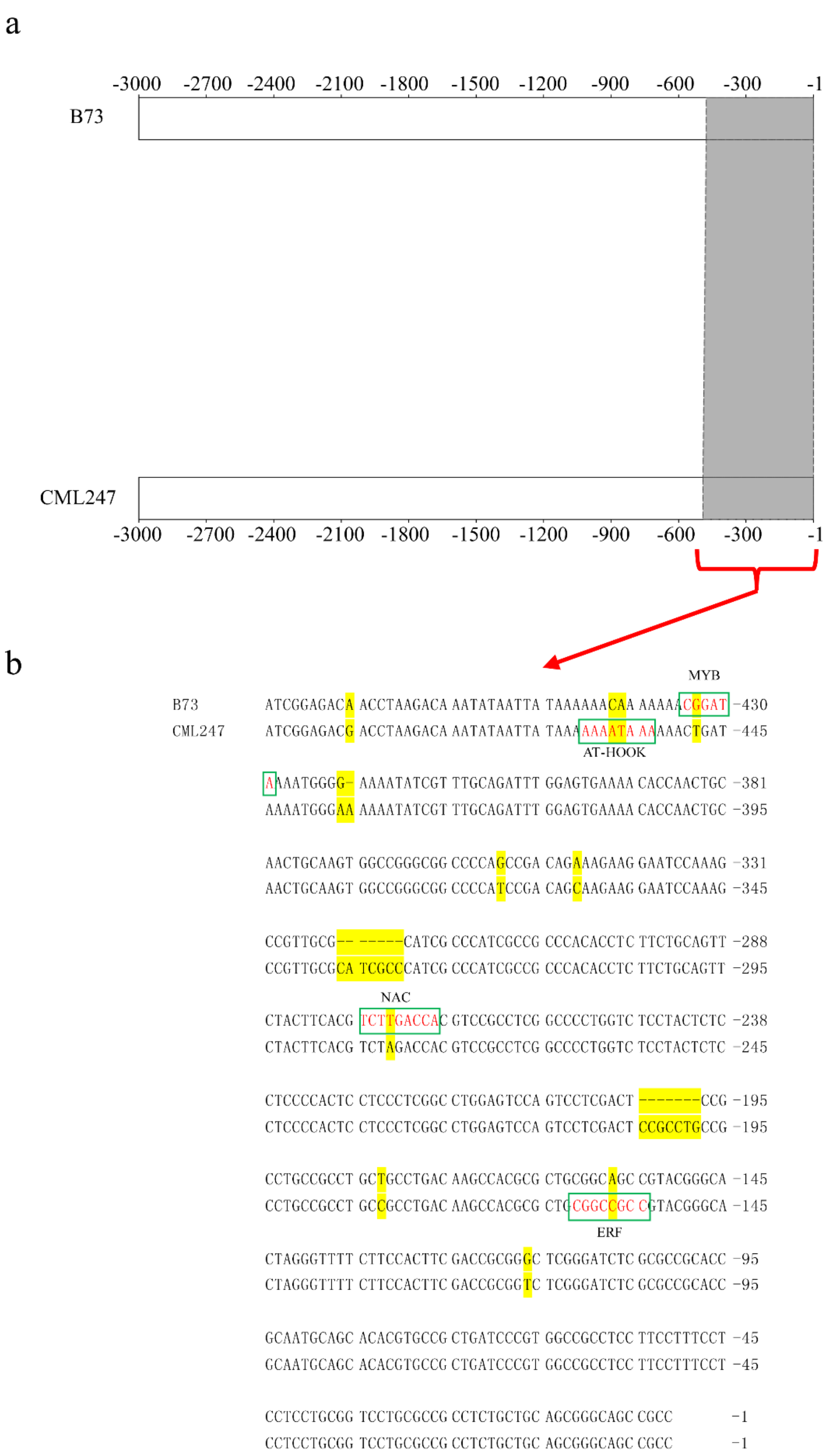

Figure S4. Analysis of sequence alignment. **a**: The *ZmPAT7* promoter sequences with a length of 3 Kb were blasted between B73 and CML247. Only about 480 bp sequences were matched. **b**: The variation (SNPs, Indels) between B73 and CML247 on the matched sequence. The yellow positions represent the variation (SNPs, Indels), and the red fonts represent *cis*-acting elements.
